# Advances in mesenchymal stem cell transplantation for the treatment of osteoporosis

**DOI:** 10.1111/cpr.12956

**Published:** 2020-11-18

**Authors:** Yuhe Jiang, Ping Zhang, Xiao Zhang, Longwei Lv, Yongsheng Zhou

**Affiliations:** ^1^ Department of Prosthodontics Peking University School and Hospital of Stomatology National Engineering Laboratory for Digital and Material Technology of Stomatology National Clinical Research Center for Oral Disease Beijing Key Laboratory of Digital Stomatology Beijing P.R. China

**Keywords:** clinical trial, mesenchymal stem cells, osteoporosis, preclinical investigation, stem cell therapy

## Abstract

Osteoporosis is a systemic metabolic bone disease with characteristics of bone loss and microstructural degeneration. The personal and societal costs of osteoporosis are increasing year by year as the ageing of population, posing challenges to public health care. Homing disorders, impaired capability of osteogenic differentiation, senescence of mesenchymal stem cells (MSCs), an imbalanced microenvironment, and disordered immunoregulation play important roles during the pathogenesis of osteoporosis. The MSC transplantation promises to increase osteoblast differentiation and block osteoclast activation, and to rebalance bone formation and resorption. Preclinical investigations on MSC transplantation in the osteoporosis treatment provide evidences of enhancing osteogenic differentiation, increasing bone mineral density, and halting the deterioration of osteoporosis. Meanwhile, the latest techniques, such as gene modification, targeted modification and co‐transplantation, are promising approaches to enhance the therapeutic effect and efficacy of MSCs. In addition, clinical trials of MSC therapy to treat osteoporosis are underway, which will fill the gap of clinical data. Although MSCs tend to be effective to treat osteoporosis, the urgent issues of safety, transplant efficiency and standardization of the manufacturing process have to be settled. Moreover, a comprehensive evaluation of clinical trials, including safety and efficacy, is still needed as an important basis for clinical translation.

## Graphical abstract

Osteoporosis occurs when the orchestrated balance between bone formation by osteoblasts and bone destruction by osteoclasts breaks down, and this process is closely associated with MSCs. Existing preclinical evidences of MSCs derived from different tissues support MSCs‐based cell therapy as a possible radical approach to the prevention and treatment of osteoporosis. Clinical trials of MSC transplantation for osteoporosis mainly focus on the application of autologous cells and will fill the gap of clinical data.

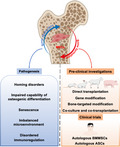

## 1 INTRODUCTION

Osteoporosis is characterized as a quantitative and qualitative deterioration of bone tissues causing increased risks of fracture.^1^ It is classified as primary (with unknown cause) and secondary (with traceable aetiology) osteoporosis. Primary osteoporosis is further classified as Type‐I post‐menopausal (between 50 and 70 years old) and Type‐II age related (more than 70 years old affecting both trabecular and cortical bone), while secondary causes of osteoporosis include hypercortisolism, hyperthyroidism, hyperparathyroidism, alcohol abuse and immobilization.^2^ Diagnosis of osteoporosis is mainly on the basis of T‐score, which reflects the bone mineral density (BMD) of lumbar vertebrae and the femoral necks. Under the unified definition of WHO, patients with a T‐score < −2.5 standard deviation (SD) of the young female adult mean are diagnosed as having osteoporosis, while those with a T‐score between −1 SD and −2.5 SD of the young female adult mean are categorized as having osteopenia.^3^ Moreover, the WHO Fracture Risk Assessment Tool (FRAX) is considered to be efficient in estimating the long‐term risk of fracture.^4^ Currently, the prevalence of osteoporosis among people over 50 years old in Europe and the United States is 4%‐6%,^5^, ^6^ while in Asia it is above 15%.^7^, ^8^ With the increasing prevalence resulting from the ageing population, osteoporosis has been recognized as a major public health concern.

The mainstream treatment of osteoporosis is to stimulate osteogenesis or inhibit bone resorption through drug‐based agents.^9^ Bisphosphonates, the predominant first‐line drugs to treat osteoporosis, decrease bone resorption by promoting osteoclast apoptosis.^10^ Alternative anti‐resorption drugs include denosumab and calcitonin.^11^, ^12^ Oestrogen and raloxifene have been applied in hormone therapy to retard the process of bone breakdown and reduce fracture risk in post‐menopausal women.^13^ Chinese medicines, such as rhizoma drynariae^14^ and icariin,^15^ have been shown to maintain BMD in osteoporosis. In addition, non‐pharmacological treatments such as vitamin D and calcium intake have also been used.^16^ However, drug‐based treatments have two obvious drawbacks: First, they cannot reverse the existing bone loss, and second, they always lead to serious side effects, including osteonecrosis of the jaw, cancer, risk of thromboembolic events, and strokes.^17^ Therefore, there is an urgent need for alternative therapeutic methods for osteoporosis.

Mesenchymal stem cells (MSCs) are a breed of undifferentiated cells with self‐proliferation and multi‐linage differentiation capabilities, which have been proven to be closely related to the progression of osteoporosis.^18^ During recent decades, MSCs are high‐profile, not only because their widespread application in basic research, but also because their potential capabilities to develop therapeutic strategies for a wide range of pathophysiological disorders in regenerative medicine.^19^ MSCs also have promising application in the treatment of osteoporosis.

In this review, we summarize the effects, mechanisms, and potential clinical applications of MSCs in the field of primary osteoporotic therapy. Meanwhile, reported progress in preclinical studies as well as several strategies aiming to enhance the therapeutic effects of MSCs is discussed. Furthermore, we introduce recent completed or ongoing clinical trials. Finally, the major obstacles to the development of MSC transplantation and future trends are discussed.

## 2 METHODS

Three databases (PubMed, MEDLINE and Web of Science) were used for primary literature collection from (January 1950‐15 May 2020). The following keywords and their combinations were used: ((Mesenchymal Stem Cells) OR (Stem Cell, Mesenchymal) OR (Stem Cells, Mesenchymal) OR (Mesenchymal Stem Cell) OR (Mesenchymal Stromal Cells) OR (Mesenchymal Stromal Cell) OR (Stromal Cell, Mesenchymal) OR (Stromal Cells, Mesenchymal) OR (Wharton Jelly Cells) OR (Wharton's Jelly Cells) OR (Wharton's Jelly Cell) OR (Whartons Jelly Cells)) AND ((Osteoporosis) OR (Osteoporoses) OR (Osteopenic) OR (bone loss) OR (bone losses)). The abstracts of the articles were screened based on the following inclusion criteria:

- Only original research articles, but not reviews, were included.
- Studies based on MSC transplantation in osteoporotic models, including the treatment of systematic osteoporosis, osteoporotic fractures, and bone defects under osteoporotic conditions, were included.

**FIGURE 1:**
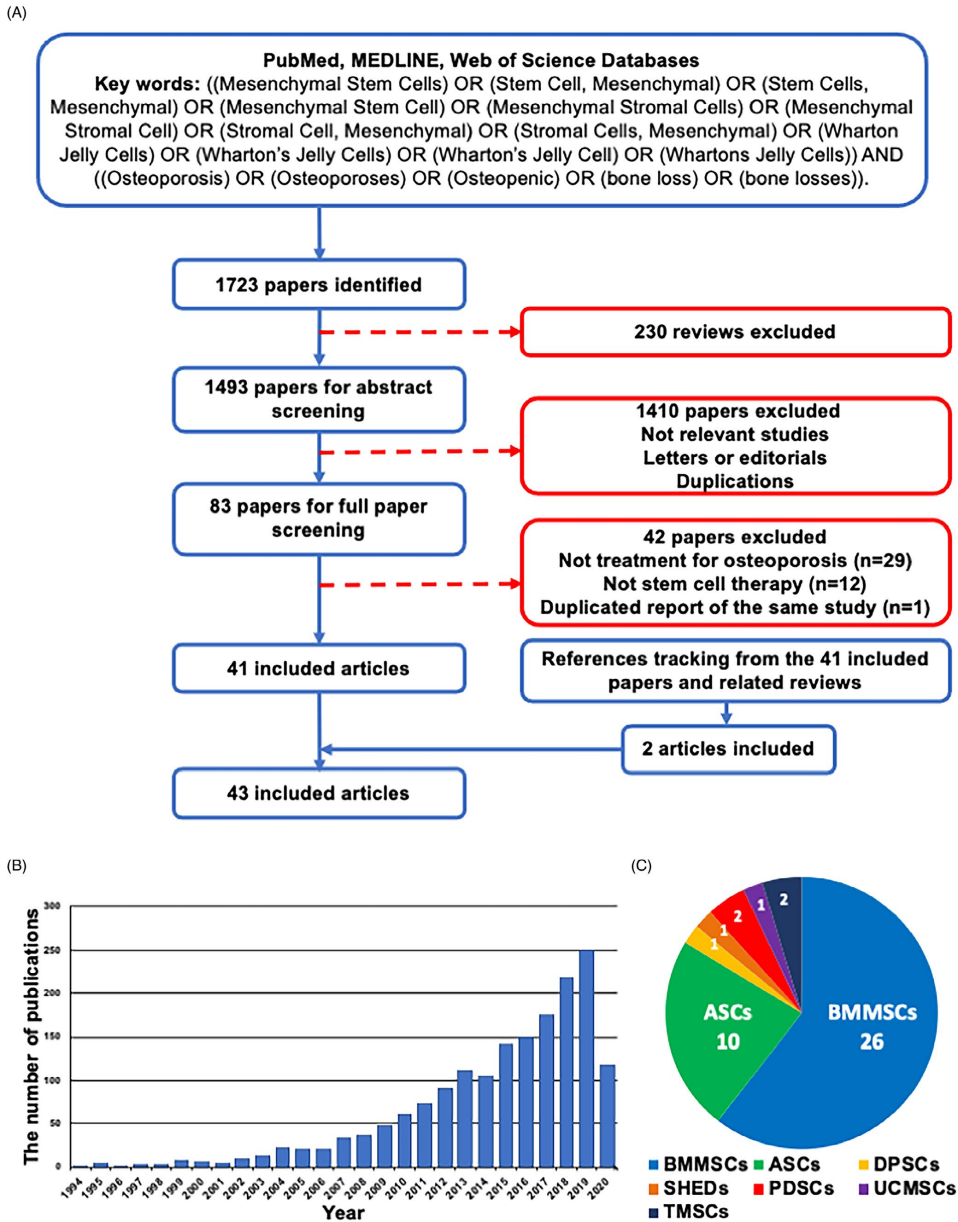
Overview of the included articles for mesenchymal stem cell (MSC) transplantation in the treatment of osteoporosis. A, Flow diagram illustrating the study screening and inclusion process. B, Statistics for the numbers of publications in different years. C, Types of cells of the included articles. ASCs, adipose‐derived mesenchymal stem cells; BMMSCs, bone marrow mesenchymal stem cells; DPSCs, dental pulp stem cells; SHEDs, stem cells from human exfoliated deciduous teeth, PDSCs, placenta‐derived mesenchymal stem cells; TMSCs, tonsil‐derived mesenchymal stem cells; UCMSCs, umbilical cord blood mesenchymal stem cells

A total of 1723 articles were retrieved after the initial search of the databases and then 230 reviews were excluded. After screening the titles and abstracts, 1410 articles were excluded mainly because they were not considered to be of relevance to the current analysis, or they were letters, editorials, or duplicate reports. Among the 83 potentially relevant studies, 42 were further excluded after reviewing the full texts because 29 studies were unrelated to the treatment of osteoporosis, 12 studies were unrelated to stem cell therapy and one paper represented repetition of the same studies. Reference tracking was performed on the full texts of the resulting articles to find missing articles that met the inclusion criteria. Two articles fulfilled the inclusion criteria. The final number of included articles was 43 (Figure 1A). During the last decade, the number of publications in this field has been increasing year by year, which indicates the research value and practical significance of cell therapy (Figure 1B). Among the included studies, bone marrow mesenchymal stem cells (BMMSCs), and adipose‐derived mesenchymal stem cells (ASCs) were the most common MSCs used to treat osteoporosis, accounting for more than three quarters of the total. Dental related MSCs and MSCs from other tissue sources have also received attention in recent years (Figure 1C).

## 3 MSCs IN THE PATHOGENESIS OF OSTEOPOROSIS

**FIGURE 2:**
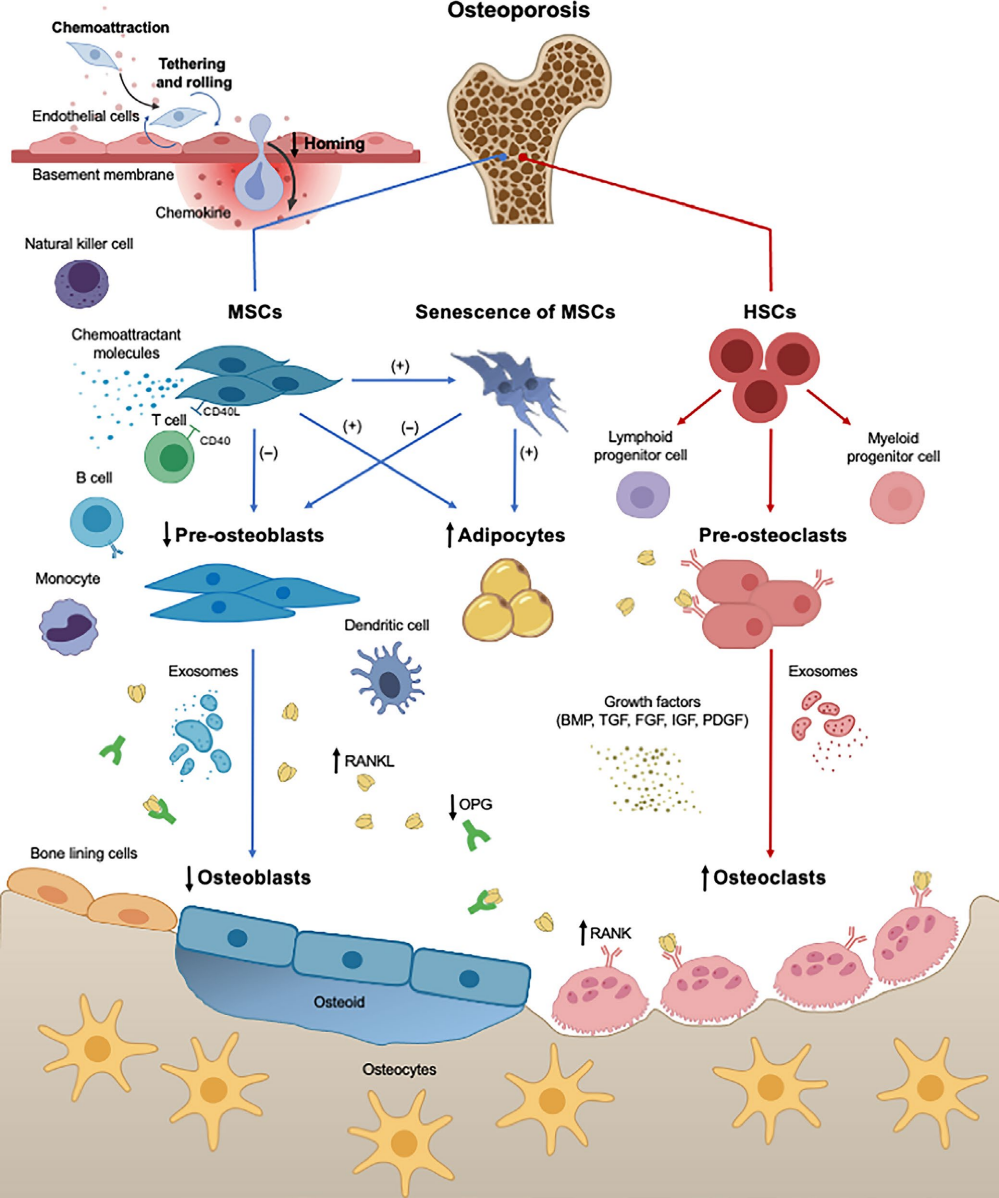
Mesenchymal stem cells (MSCs) in the pathogenesis of primary osteoporosis. Homing disorder results in a decreased number of MSCs in bone tissue under osteoporotic conditions. Impaired osteogenic ability and enhanced adipogenic ability of MSCs leads to less mature osteoblasts and more adipocytes. Senescence of MSCs further aggravates the imbalance of osteoblasts and adipocytes. In addition, abnormal activation of immune cells and impaired immunoregulatory ability of MSCs causes immune disorders in the bone niche, with altered cellular interactions and imbalanced paracrine secretion of many key signalling factors, such as RANK‐RANKL‐OPG axis. ↑indicates an increase in the number of cells/factors.↓indicates a decrease in the number of cells/factors. (+) represents the enhancement of the process. (−) represents the inhibition of the process. BMP2, bone morphogenetic protein 2; FGF, fibroblast growth factor; HSCs, hematopoietic stem cells; IGF, insulin‐like growth factor; OPG, osteoprotegerin; PDGF, platelet‐derived growth factor; PPARγ, peroxisome proliferator‐activated receptor γ; RANK, receptor activator of NF‐κB; RANKL, receptor activator of NF‐κB ligand

The pathogenesis of primary osteoporosis is generally recognized as the imbalance between bone formation and resorption during bone reconstruction, in which the speed of bone absorption is greater than that of bone formation, leading to increased bone turnover. Homing disorders, impaired capability of osteogenic differentiation, and senescence of MSCs are important pathogeneses of primary osteoporosis. An imbalanced microenvironment and disordered immunoregulation also have key impacts on the occurrence and development of osteoporosis (Figure 2).

### 3.1 Homing disorders

Homing is the first step of bone repair, in which MSCs migrate to bone marrow to exert a local functional and restorative role. Common knowledge is that MSCs follow similar steps to leukocyte homing.^20^ The first step is the cells contact with the endothelium by tethering and rolling, bringing about the cells decelerating in bloodstream. The second step is the activation of cells by G‐protein coupled receptors, and integrin‐mediated, activation‐dependent arrest come next in the third step. The last step is the cells migrate through endothelial cells and underlying basement membrane.

In the case of reduced homing ability, it is difficult to ensure that enough MSCs can reach the damaged tissue, which hinders bone repair.^21^ Sanghani et al^22^ showed that both ageing and osteoporosis impaired MSC migration, and this might be referable to a significant reduction in bone formation in patients with osteoporosis. More importantly, their study emphasized the positive effect of C‐X‐C motif receptor 4 (CXCR4) overexpression on MSC migration. Haasters et al^23^ found MSCs from patients with osteoporosis showed a surge in the migration upon bone morphogenetic protein 2 (BMP‐2) stimulation, as well as their invasion increased significantly upon BMP‐2 or BMP‐7 stimulation. Nevertheless, the invasion and migration capacity decreased significantly compared with those of the healthy controls. Therefore, increasing the total number of MSCs through cell transplantation or enhancing the homing of MSCs through gene modification or targeted peptides would be helpful to solve this problem.

### 3.2 Impaired capability of osteogenic differentiation

Common mesenchymal progenitor cells differentiate into various types of skeleton‐related cells is determined by multiple transcription factors and signalling pathways. The initial step in osteoblastic differentiation is the determination of a MSC to become an osteoprogenitor, in which mesenchymal progenitor cells are directed to preosteoblasts by runt‐related transcription factor 2 (RUNX2), while chondrocyte and adipocyte differentiation are inhibited.^24^ Next, RUNX2 and Osterix (OSX) guide preosteoblasts to immature osteoblasts expressing bone matrix protein genes, completely eliminating the potential for chondrocytic differentiation.^25^ Furthermore, the BMP signalling pathway is generally acknowledged to play important roles in regulating the adipogenic and osteogenic differentiation of MSCs.^26^ BMP‐2 accelerates the osteogenic differentiation of stem cells.^27^ However, BMP‐2 can act as a potent adipogenic agent if presented together with activators of peroxisome proliferator‐activated receptor γ (PPARγ).^28^

The reduction of osteogenic differentiation is the core of osteoporosis. Rodriguez et al^29^ detected MSCs from patients with osteoporosis that produced a type‐I collagen‐deficient extracellular matrix which favoured adipogenic differentiation in the preliminary stage. Wang et al^30^ compared the MSCs of post‐menopausal women with osteoporosis and healthy volunteers, and confirmed that the sensitivity of MSCs to osteogenic differentiation was decreased in patients with osteoporosis. Pino et al^31^ found that in patients with osteoporosis, the osteogenic differentiation of MSCs was weakened, while adipogenic differentiation was strengthened, leading to a decline in bone formation and the accumulation of marrow adipose tissue (MAT). In order to reverse this trend, MSCs with better osteogenic differentiation ability should be transplanted. Reactivated the osteogenic differentiation ability of MSCs by gene modification and in vitro activator is also a feasible way.

### 3.3 Senescence

Osteoporosis is also associated with the senescence of MSCs. Zhou et al^32^ discovered that the number of MSCs in elderly patients with osteoporosis was much lower than that in young people; the doubling time in MSCs from the older was 1.7‐fold longer than those from the younger subjects, and the content of β‐galactosidase related to ageing was four times that of young people. Stolzing et al^33^ conducted in vitro passage culture of MSCs from 57 volunteers aged 5‐55 years, and the results showed that the proliferative ability and cell activity of MSCs decreased with age, accompanied by weakened osteogenic differentiation and relatively enhanced adipogenic differentiation. At present, gene modification is an effective strategy to delay the senescence of MSCs.

### 3.4 Imbalanced microenvironment

Bone remodelling is a complex coordinated event requiring various cell types to activate synchronously in the microenvironment to ensure that both bone formation and bone resorption occurs successively to sustain bone mass.^34^ This process starts at the initiation stage by activating osteoclasts under the regulation of osteoclastogenic factors, including receptor activator of NF‐κB ligand (RANKL) and macrophage colony‐stimulating factor (M‐CSF),^35^ followed by osteoblast‐mediated bone formation. In this process, exosomes are regarded as paracrine regulators. The number of mature phenotypes differentiate from osteoclasts stimulated by osteoclast precursor‐derived exosomes is significantly larger than that in the absence of exosomes.^36^ Nevertheless, osteoblast‐derived exosomes which contain RANKL can arouse osteoclast formation by activating RANK signalling in osteoclast precursors through the RANKL‐RANK interaction.^37^ Xu et al^38^ reported the existence of microRNAs (miRNAs) in exosomes during BMMSC osteogenic differentiation, which have been proven to repress adipogenesis and activate osteogenesis by enhancing key osteoblast signalling molecules. Moreover, this cycle is in the charge of bone lining cells and osteocytes.^39^ Several coupling factors, including BMP, transforming growth factor β (TGF‐β), fibroblast growth factor (FGF), insulin‐like growth factor (IGF) and platelet‐derived growth factor (PDGF), are also involved in the process.^40^, ^41^ Dalle et al^42^ found a lower OPG (osteoprotegerin)/RANKL ratio in the supernatants of osteoblastic culture from patients with osteoporosis than that from normal donors, which caused an alteration of osteoblastic differentiation and might contribute to the pathogenesis of osteoporosis. Abnormal miRNA levels are also involved in the occurrence of primary osteoporosis through regulating osteoclast and osteoblast differentiation.^43^, ^44^ Therefore, disorders of important factors and signalling pathways regulating MSC differentiation in the microenvironment may cause an imbalance of bone metabolism, eventually leading to osteoporosis. Exogenous MSC transplantation is expected to redress the imbalance of microenvironment by regulating related factors and signalling pathways through paracrine.

### 3.5 Disordered immunoregulation

Recently, the close relationship between bones and the immune system has been recognized, particularly when both systems are activated under pathological conditions.^45^ Immune cells can influence bone‐related cells by the secretion of various immune factors. Nearly all the subtypes of T cells can influence bone cells. Among them, the important roles of T‐helper(Th) 17 and regulatory T cells (Tregs) in the regulation of osteoclast activity have been noted. Th17 cells have been proved to induce the expression of M‐CSF and RANKL in osteoblasts and MSCs, and increase the expression of RANK in osteoclast precursors, leading to excessive activation of osteoclasts.^46^, ^47^ With regards to Treg cells, their effect has been recognized to suppress osteoclast formation.^48^ D’Amelio et al^49^ found a significant increase in tumour necrosis factor α (TNF‐α) produced by T cells and monocytes derived from osteoporotic post‐menopausal patients, which stimulates osteoclast formation in bone loss induced by oestrogen deficiency. Dendritic cells (DCs) and natural killer cells (NKs) also participate in osteoclastogenesis by regulating the subtype balance and activity of T cells through cytokine signalling.^50^, ^51^

On the other hand, MSC‐mediated osteoimmunology was also altered under osteoporotic conditions. Available evidence suggests that MSCs may stimulate the differentiation of Treg cells, and induce the apoptosis of the pro‐inflammatory Th1 and Th17 cells.^52^, ^53^ Meanwhile, Corcione et al^54^ found MSCs could inhibit migration of B cells to exert an immunosuppressive role in bone repair by downregulating the expression of chemokine receptors and their ligands. In addition, MSCs can also affect monocytes, DCs and NKs by secreting chemoattractant molecules.^45^ Therefore, the interaction between immune cells and MSCs is paramount to bone metabolism, and the abnormal levels of inflammatory factors lead to the excessive activation of osteoclasts, leading to pathologic bone destruction and bone loss.

## 4 PRECLINICAL INVESTIGATIONS

**FIGURE 3:**
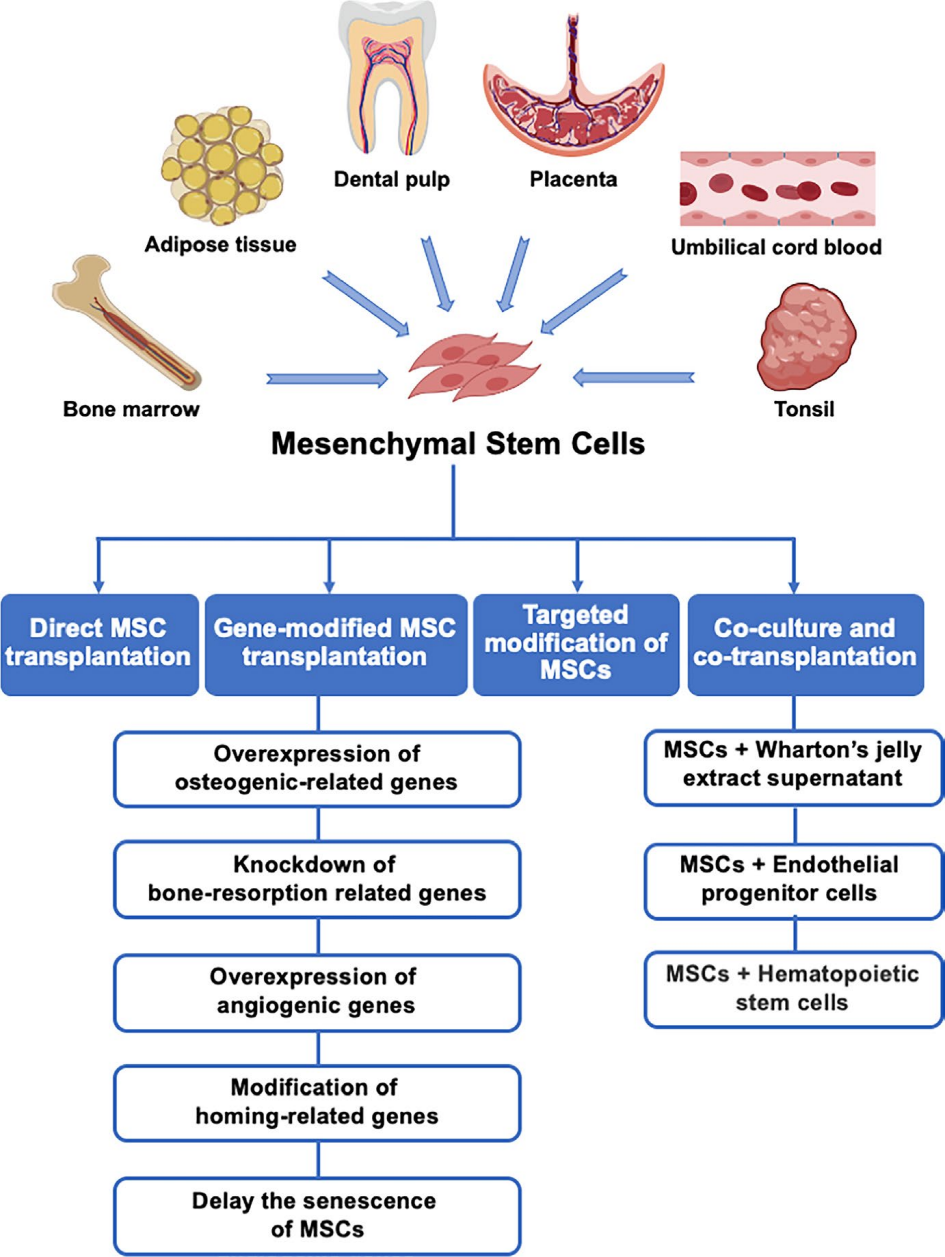
Preclinical studies on mesenchymal stem cell (MSC) transplantation in the treatment of osteoporosis

MSCs can be insulated from amount of tissues (eg bone marrow, dental pulp, adipose tissue, umbilical cord, placenta and tonsil) and selective cultured prior to clinical use. According to their capacity to differentiate towards multiple mesenchymal lineages, MSCs have shown promises for wide applications in regenerative medicine and tissue engineering. Intra‐bone marrow and intra‐tail venous injections are common methods for MSC transplantation to treat osteoporosis (Figure 3).

### 4.1 Direct MSC transplantation

Direct MSC transplantation has long been the focus of researchers, and the results based on osteoporotic animal models are relatively mature (Table 1). BMMSCs and ASCs have been widely used in ovariectomized (OVX) osteoporotic and age‐related osteoporotic models, and their effects on promoting osteogenic differentiation have been verified repeatedly. Meanwhile, dental mesenchymal stem cells improve OVX‐induced osteoporosis mainly through paracrine and immune regulation. For placenta‐derived mesenchymal stem cells (PDSCs) and umbilical cord blood mesenchymal stem cells (UCMSCs), the mechanisms of osteoporosis treatment are mainly reflected in improved osteoblast activity and the weakening of osteoclast differentiation. Tonsil‐derived mesenchymal stem cells (TMSCs) simultaneously enhance osteogenic differentiation and block MAT accumulation.

**TABLE 1:** Preclinical studies of direct MSC transplantation for osteoporosis

Cell type	Animal model	Stem cell origin	Delivery method	Therapeutic outcomes	Author, year, reference
BMMSCs	OVX osteoporotic rats	Allogeneic	IBM	Promoted trabecular reconstruction and improved bone quality	Yu et al, 2012^55^
BMMSCs	OVX osteoporotic rats	Allogeneic	IBM	Increased trabecular bone, attenuated the loss of BMD, and improved the femur bone mass	Ocarino et al, 2010^56^
BMMSCs	OVX osteoporotic rats	Allogeneic	IBM	Improved BMD, ultimate load, and stiffness of in the BMMSC‐injected bones	Uejima et al, 2008^57^
BMMSCs	OVX osteoporotic mice	Allogeneic	ITV	Improved BMD, trabecular volume, and trabecular number to some extent	Yang et al, 2013^58^
BMMSCs	OVX osteoporotic rabbits	Autologous	IBM	Improved microstructures, and enhanced the trabecular thickness and stiffness of bone	Wang et al, 2006^59^
BMMSCs	OVX osteoporotic goats	Autologous	IBM	Repaired osteoporotic bone defects successfully by combining autologous enriched BMMSCs with β‐TCP	Cao et al, 2012^60^
BMMSCs	Age‐related osteoporotic mice	Allogeneic	ITV	Improved bone quality and turnover, and sustained microarchitectural competence	Kiernan et al, 2016^61^
BMMSCs	Age‐related osteoporotic mice	Allogeneic	IBM	Increased trabecular bone, attenuated the loss of BMD, improved the femur bone mass	Ichioka et al, 2002^62^
ASCs	OVX osteoporotic rats	Autologous	IBM	Increased mean cortical thickness, total bone volume density, and bone load to failure significantly	Uri et al, 2018^63^
ASCs	OVX osteoporotic rats	Allogeneic	ITV	Improved BMD, bone trabecular absorption surface percentage, and the rate of bone trabecular formation	Li et al, 2016^64^
ASCs	OVX osteoporotic mice	Xenogenic (human)	ITV	Protected against ovariectomy‐induced attenuation in bone mass gain	Cho et al, 2012^65^
ASCs	OVX osteoporotic rabbits	Autologous	IBM	Increased BMD and formed more new bone in the cell‐treated femurs	Ye et al, 2014^66^
ASCs	Age‐related osteoporotic mice	Allogeneic	IBM	Restored BMD in the knees, femurs and spine	Liu et al, 2012^67^
ASCs	Age‐related osteoporotic mice	Allogeneic	IBM	Improved trabecular bone quality and increased molecular markers of bone turnover	Mirsaidi et al, 2014^68^
ASCs	OVX osteoporotic mice	Allogeneic	IBM	ASCs from osteoporotic donors maintain efficacy to hold bone remodelling balance	Zheng et al, 2018^69^
DPSCs	OVX osteoporotic mice	Xenogenic (human)	IBM	Reduced OVX‐induced bone loss in the trabecular bone of the distal femur metaphysis significantly	Kong et al, 2018^70^
SHEDs	OVX osteoporotic mice	Xenogenic (human)	ITV	Rescued BMMSC deficiency and ameliorated the osteopenia phenotype in OVX mice	Liu et al, 2014^71^
PDSCs	OVX osteoporotic rats	Xenogenic (human)	IBM	Increased the rod‐shaped trabecular bone and the accumulation of collagen	Fu et al, 2018^72^
PDSCs	OVX osteoporotic rats	Xenogenic (human)	ITV	Formed new bone trabeculae and reduced the damage to trabecular structure	Lei et al, 2017^73^
UCMSCs	OVX osteoporotic mice	Xenogenic (human)	ICV	Augmented bone formation rate, BMD and improved bone micro‐architecture	Aggarwal et al, 2012^74^
TMSCs	OVX osteoporotic mice	Xenogenic (human)	IBM	Recovered serum osteocalcin level and reduced visceral fat	Kim et al, 2018^75^
TMSCs	Age‐related osteoporotic mice	Xenogenic (human)	ITV	Sustained osteocalcin production and blocked MAT accumulation	Kim et al, 2016^76^

Abbreviations: ASCs, adipose‐derived mesenchymal stem cells; BMD, bone mineral density; BMMSCs, bone marrow mesenchymal stem cells; DPSCs, dental pulp stem cells; IBM, intra‐bone marrow; ICV, intra‐cardio ventricular; ITV, intra‐tail venous; MAT, marrow adipose tissue; OVX, ovariectomized; PDSCs, placenta‐derived mesenchymal stem cells; SHEDs, stem cells from human exfoliated deciduous teeth; TMSCs, tonsil‐derived mesenchymal stem cells; UCMSCs, umbilical cord blood mesenchymal stem cells.

BMMSCs have been extensively investigated in bone regeneration and repair because of their osteogenic differentiation capacity.^21^ A number of preclinical investigations implied that BMMSC transplantation in OVX model animals (eg rats,^55^, ^56^, ^57^ mice,^58^ rabbits^59^ and goats^60^) could help to strengthen osteoporotic bones resulting from oestrogen deficiency: (a) Bone density increased significantly, indicating that bone destruction and loss could be reversed to some extent; (b) trabecular volume, trabecular number, trabecular thickness, percentage of trabecular area and trabecular spacing were increased, indicating that microstructural degeneration could be alleviated to some extent; and (c) the levels of osteogenic markers in serum, such as calcium, alkaline phosphatase (ALP) and osteocalcin (OCN), increased after MSC injection. Furthermore, Uejima et al^57^ and Wang et al^59^ injected BMMSCs into distal femurs and evaluated their mechanical properties using biomechanical testing, illustrating that BMMSCs aided the preservation of mechanical properties. Yu et al^58^ found after BMMSCs transplantation, the level of TNF‐α decreased, while T‐cell apoptosis, BMD, trabecular number, and bone volume fraction increased. This suggested that BMMSCs might play a critical role in treating oestrogen deficiency‐induced osteoporosis through immunoregulation of the apoptosis of T cells. Meanwhile, Kiernan et al^61^ and Ichioka et al^62^ observed long‐term engraftment and significant increased bone formation in age‐related osteoporosis after MSC transplantation. Therefore, BMMSC transplantation is likely to be a feasible therapeutic strategy to prevent or treat both oestrogen‐deficient and age‐related osteoporosis.

ASCs have the advantages of easy accessibility, less donor site morbidity, satisfactory proliferative capacity and the ability to differentiate into multilineage cells, including osteoblasts and adipocytes.^77^ In the cell therapy of osteoporosis, ASCs have been reported as effective autologous cells. The mechanism of improving OVX‐induced osteoporosis is similar to that of BMMSCs, which is mainly reflected in three aspects: (a) Significant increases in cortical thickness, bone volume density and bone load^63^, (b) improved trabecular microstructure^64^, and (c) increased serum calcium and OCN levels.^65^ Ye et al^66^ revealed that osteogenic‐induced ASCs promoted osteogenesis and inhibited adipogenesis of osteoporotic BMMSCs by activating BMP‐2 signalling pathway, which explained above as an important pathway in osteogenic differentiation. Liu et al^67^ and Mirsaidi et al^68^ also documented the effectiveness of ASCs transplantation in mice with age‐related osteoporosis. As mentioned earlier, an imbalanced microenvironment also plays important role in the pathogenesis of osteoporosis. Despite the imbalanced microenvironments and related systemic inflammation of OVX donors, ASCs preserved anti‐inflammatory capacity and continued to safeguard bone formation in OVX recipients. However, BMMSCs from OVX‐induced osteoporotic mice failed to restrain bone loss after being infused back into OVX recipients for the incapacitation of anti‐inflammatory.^69^ Meanwhile, Chen et al^78^ proved that ageing and passaging had less effect on the proliferation and osteogenic differentiation of ASCs compared with that on BMMSCs. Liu et al^67^ also verified that mice receiving young ASCs showed markedly higher osteogenesis (an average of 24.3% improved BMD) than those receiving aged ASCs. Therefore, ASCs are an encouraging therapeutic option for the osteoporosis treatment, because autologous ASCs preserve their anti‐inflammatory capacity under the general osteoporotic conditions compared with BMMSCs and are less affected by in vitro passaging.

Dental mesenchymal stem cells have aroused research interests since their early discovery. Studies focusing on stem cells from the dental pulp of permanent and deciduous teeth to regenerate or repair non‐dental tissues have proved their effectiveness in bone, skin, nervous tissue, and vascular tissue regeneration.^79^ Dental pulp stem cells (DPSCs) were reported to have a higher osteogenic capability compared with that of BMMSCs, and their adipogenic potential was found to be weaker than that of BMMSCs.^80^ Kong et al^70^ reached the following conclusions: (a) Some DPSCs could survive for more than 1 month in vivo. (b) DPSCs had higher homing efficiency when transplanted in the early period of OVX mice. (c) After administration, DPSCs were mainly distributed to the lung first and then in the liver; however, they were barely distributed to the bone. (d) Mediated by paracrine mechanisms, DPSC transplantation reduced OVX‐induced bone loss in trabecular bone of distal femur metaphysis significantly, suggesting systemic infusion of DPSCs was a potential treatment for OVX‐induced osteoporosis. Another multipotent stem‐cell population found in remnant dental pulp derived from exfoliated deciduous teeth are defined as stem cells from human exfoliated deciduous teeth (SHEDs).^81^ SHEDs are a peculiar postnatal stem cell population characterized by multipotent differentiation capacity and immunoregulation properties, thus cryopreserved dental pulp tissues of exfoliated deciduous teeth are considered as practicable stem cell resources for regenerative medicine.^82^ They relieve osteoporosis mainly through immunoregulation. Liu et al^71^ showed that systemic injection of SHEDs via the tail vein ameliorated OVX‐induced osteopenia by activating the Fas ligand (FasL)‐mediated Fas pathway, leading to up‐regulation of Tregs and down‐regulation of Th1 and Th17 cells. This SHED‐mediated immunoregulation increased bone mass by rescuing OVX‐induced impairment of BMMSCs and inhibiting osteoclast differentiation. However, SHEDs and DPSCs show different expression levels of the osteoblast markers for osteoblastic differentiation, suggesting that SHEDs exhibit a higher capacity for osteogenic differentiation in comparison with DPSCs and are noticeably different to DPSCs.^83^ Furthermore, at early and late passages, SHEDs exhibit higher multiplication and osteogenic differentiation capacity compared with those of DPSCs.^84^ Therefore, dental mesenchymal stem cells, such as DPSCs and SHEDs, could also be feasible alternatives in the treatment of osteoporosis.

PDSCs are another abundant source of stem cells that have unique inherent characteristics.^85^ Experimental evidence in OVX rats^72^, ^73^ indicated that human PDSCs had therapeutic effects on OVX‐induced osteoporosis. In addition to increased bone density, alleviated microstructural degeneration, and increased OCN and ALP levels in the serum, the transplantation of PDSCs also improved osteoblast activity and simultaneously weakened osteoclast differentiation, maturation, and functionality.^72^ Further research suggested that transplantation of PDSCs might promote the expression of RUNX2 and OSX to achieve this effect.^73^ However, the use of PDSCs always elicits political, ethical, moral and emotional debate over their application in research. Human UCMSCs do not need myeloablation for efficacy or to overcome allogeneic barriers to cellular therapies with banked cord blood.^86^ Aggarwal et al^74^ verified significant improvements in bone deposition, BMD and bone micro‐architecture after delivering UCMSCs systemically to the bone marrow in osteoporotic mice. The elevated levels of OCN in serum paralleled the advancements in bone micro‐architecture. Moreover, UCMSCs improved osteoblast activity and impaired osteoclast differentiation, maturation and functionality in the meantime.

TMSCs, isolated from tonsils, have the potential to differentiate not only into the mesodermal lineage, but into the endodermal and ectodermal lineages, extending their potential use and identifying them as a fascinating option to consider for future investigations in cell therapy.^87^, ^88^ Kim's^75^ results showed that double injection of TDSCs directly into the proximal tibia recovered serum OCN levels and triggered recovery of osteoporosis. In age‐related osteoporotic mice, Kim et al^76^ injected TDSCs via the tail vein. The results demonstrated that TDSCs attenuated the progression of osteoporosis partially, not only by sustaining OCN production, but also by blocking MAT accumulation. Regulation of MAT together with bone could be considered to be a new therapeutic approach for the treatment of age‐related osteoporosis.

### 4.2 Gene‐modified MSC transplantation

To achieve improved osteogenic and angiogenic capabilities of transplanted cells, gene modification of important osteogenic and/or angiogenic genes has been taken into consideration before MSC transplantation. According to the included research, in general, five strategies have been used (Table 2): (a) Overexpression of osteogenic‐related genes, including *BMP‐2*, *BMP‐6*, *RUNX2* and *OSX*; (b) knockdown of genes for bone destruction, such as receptor activator of nuclear factor‐κB‐Fc (*Rank‐fc*), to inhibit osteoclast activation; (c) overexpression of angiogenic genes, such as encoding fibroblast growth factor 2 (*FGF2*) and encoding platelet‐derived growth factor subunit B (*PDGFB*), to favour angiogenesis, thus promoting osteogenesis; (d) modification of homing‐related genes, such as activating *CXCR4*, to heighten homing and migration of MSCs; and (e) efforts to delay the senescence of MSCs, such as activating telomerase reverse transcriptase (*TERT*) to prolong or stabilize telomeres.

**TABLE 2:** Preclinical studies of gene‐modified MSC transplantation for osteoporosis

Target gene	Vector	Cell type	Animal model	Stem cell origin	Transplant method	Therapeutic outcomes	Author, year, reference
*BMP‐2*	NR	ASCs	OVX osteoporotic rats	Allogeneic	ITV	Increased the continuity of the trabecular bone with less widened spacing than control groups	Yang et al, 2020^89^
*BMP‐2*	Baculovirus	ASCs	OVX osteoporotic rats	Allogeneic	IBM	Further synergized the OVX‐ASC‐mediated bone regeneration	Li et al, 2017^90^
*BMP‐2*	Plasmids	BMMSCs	OVX osteoporotic rats	Allogeneic	IBM	Completely closed the osteoporotic defect and the newly formed mature bone had a typical trabecular pattern	Tang et al, 2008^91^
*BMP‐2*	Plasmids	BMMSCs	OVX osteoporotic rats	Allogeneic	IBM	New bone formed and later appeared mature	Tang et al, 2006^92^
*BMP‐2*	Adenovirus	BMMSCs	OVX osteoporotic sheep	Allogeneic	IBM	Formed larger cross‐sectional callus area and higher callus density	Egermann et al, 2006^93^
*BMP‐6*	Plasmids	BMMSCs	OVCF minipigs	Allogeneic	IBM	Regenerated the vertebrae architecture almost completely	Pelled et al, 2016^94^
*BMP‐6*	Lentivirus	ASCs	OVCF rats	Xenogenic (pig)	IBM	Increased the rate of bone formation and bone volume	Sheyn et al, 2011^95^
*Osx*	Retrovirus	BMMSCs	Age‐related osteoporotic mice	Allogeneic	IBM	Stimulated osteoblast differentiation and new bone formation, but inhibited adipocyte differentiation	Lee et al, 2014^96^
*Rank‐fc*	Retrovirus	BMMSCs	OVX osteoporotic mice	Allogeneic	IP	Attenuated bone loss in the OVX model	Kim et al, 2006^97^
*CXCR4*	Adenovirus	BMMSCs	OVX osteoporotic rats	Allogeneic	ITV	Migrated to the bone marrow more effectively and improved bone density and architecture	Sanghani et al, 2018^98^
*Cxcr4+* *Rank‐fc*	Retrovirus	BMMSCs	OVX osteoporotic mice	Allogeneic	ITV	Promoted increased in vivo cell trafficking to bone and protected against bone loss	Cho et al, 2009^99^
*PGDFB*	Lentivirus	BMMSCs	OVX osteoporotic mice	Allogeneic	ITV	Promoted proliferation of MSCs and angiogenesis	Chen et al, 2015^100^
*Tert*	Lentivirus	BMMSCs	OVX osteoporotic rats	Allogeneic	NR	Improved MSC proliferation and osteogenic differentiation ability and increased both bone mass and bone density	Li et al, 2015^101^

Abbreviations: ASCs, adipose‐derived mesenchymal stem cells; BMD, bone mineral density; BMMSCs, bone marrow mesenchymal stem cells; BMP, bone morphogenetic protein; CXCR4, C‐X‐C motif receptor 4; IBM, intra‐bone marrow; IP, Intra‐peritoneal; ITV, intra‐tail venous; NR, not reported; OSX, Osterix; OVCFs, osteoporotic vertebral compression fractures; OVX, ovariectomized; PGDFB, platelet‐derived growth factor B; RANK, receptor activator of NF‐κB; TERT, telomerase reverse transcriptase.

The most common strategy involves overexpression of osteogenesis‐related genes. BMPs, affiliated with the TGF‐β superfamily, exhibit high osteogenic activity and can stimulate the osteogenic differentiation of MSCs in vitro and in vivo.^102^ BMP‐2 is the most commonly studied. Both early and recent studies showed that MSCs, including BMMSCs^103^, ^104^ and ASCs,^105^ showed restored osteogenic activity following *BMP‐2* transduction. Further research indicated that *BMP‐2* gene transduction could restore the osteogenic potential of MSCs, which might provide a useful method in the future planning of cell and/or gene therapy for primary osteoporosis^89^, ^106^ and osteoporotic bone defects.^90^, ^91^, ^92^, ^93^ Other BMPs have also been reported for osteogenesis. Pelled et al^94^ and Shyen et al^95^ showed that MSCs overexpressing *BMP‐6* were capable of inducing spinal fusion in vivo, which could be used to treat osteoporotic vertebral compression fractures (OVCF). RUNX2 is a key transcriptional regulator that determines the fate of osteoblasts.^107^, ^108^ Expression of RUNX2 in osteochondral progenitors inhibits chondrogenic differentiation to enhance osteoblastic differentiation.^25^ Conversely, inhibition of RUNX2 prevents MSCs from differentiating into osteoblasts.^109^ OSX, a member of specificity protein 1 family (Sp1) of transcription factors with three zinc finger motifs, acts as a downstream factor of RUNX2.^110^ The expression of *RUNX2* plays a role at the initial differentiation stage, while *OSX* guarantees the complete differentiation of osteoblasts in the late stage.^25^ Its inactivation impedes osteoblast differentiation and new bone formation.^111^ Lee et al^96^ found that nuclear factor I C (encoded by *Nfic* in mice) plays a transcriptional switch role in cell fate determination between osteoblast and adipocyte in BMMSCs by downregulating *Osx* expression. It is noteworthy that transplantation of *Nfic*‐expressing BMMSCs stimulated osteoblast differentiation and bone formation, and inhibited adipocyte differentiation in *Nfic^‐/‐^* mice that showed an age‐related osteoporosis‐like phenotype.

The second strategy is to inhibit osteoclast activation by blocking osteoclastogenic factors. RANK‐Fc, a recombinant RANKL antagonist, blocks receptor activator of nuclear factor ligand specifically.^112^ Under normal circumstances, RANKL promotes osteoclast differentiation and maturation, while RANK‐Fc inhibits bone resorption by binding to RANKL to reduce the activation of osteoclast precursors.^113^ Kim et al^97^ validated whether the engraftment of *Rank‐fc* producing MSCs into bone produced bone‐protective effect. Their data demonstrated that MSC‐based gene therapy with *Rank‐fc* distinctly prevented bone resorption of OVX mice.

Angiogenesis is an essential step before new bone formation; therefore, angiogenic genes could be modified to enhance the efficacy of transplanted MSCs. PDGFB is believed to mobilize and induce migration of MSCs or osteoblasts,^114^ orchestrate cellular components for osteoblast differentiation,^115^ and stabilize newly formed vessels.^116^ Chen et al^100^ proved that *FGF2*‐modified and *PDGFB*‐modified MSCs could increase trabecular bone formation and trabecular connectivity, decrease cortical porosity, and increase bone strength by 45%. There are several possible mechanisms for the effectiveness of these two growth factors, such as: (a) BMMSC proliferation, (b) HSC proliferation, and (c) angiogenesis that is essential for bone formation. Meanwhile, synergistic promotion of osteogenic differentiation and vascularization has also been reported. Kumar et al^117^ transduced BMMSCs ex vivo with *BMP‐2* and *VEGF* and transplanted them systemically into a mouse model of segmental bone defect. Results indicated that bone formation in the MSCs‐received group was enhanced and the therapeutic effects were along with increased vascularity, and osteoblastogenesis. Chen et al^118^ isolated ASCs from minipigs and transfected them with recombinant human *BMP‐2* and *VEGF* plasmids, respectively. Subsequently, the *BMP‐2 + VEGF*‐expressing MSCs effectively repaired bone defects of the ulna in the minipigs.

The fourth strategy is to enhance the homing of MSCs. CXCR4, a specific receptor for CXCL12, is an important signalling factor in MSCs homing.^119^ CXCL12‐CXCR4 signalling is also indispensable to maintaining the hematopoietic stem cell (HSC) pool in adult bone marrow, which is closely related to osteogenesis and angiogenesis.^120^ Sanghani et al^98^ harvested MSCs from young and OVX animals. These cells were transfected with *CXCR4* cDNA and administered intravenously in OVX rats. At 12 weeks after injection, the results of micro‐computed tomography (CT) and mechanical testing revealed that rats injected with young *CXCR4*‐overexpressed cells had a significantly higher BMD. Meanwhile, strategies to promote osteogenic differentiation and enhance homing could be used in combination to enhance efficacy. Cho et al^99^ elucidated that intravenous transplantation of autologous *Cxcr4*‐overexpressing MSCs increased the homing of transplanted cells to bone in OVX mice, which could prevent bone loss and enhance the therapeutic effects of *Rank‐fc*.

Senescence of MSCs acts as an indispensable part in the pathogenesis of osteoporosis, therefore, efforts to delay senescence might be an alternative method to enhance the efficacy of transplanted cells. Telomerase can activate, prolong or stabilize telomeres, which are progressively shortened with cell division, proliferation and ageing. However, the expression of telomerase reverse transcriptase (TERT) is very low in MSCs, which limits telomerase activity and results in the senescence of MSCs.^121^ *TERT* gene knockout decreased the osteogenic ability of BMMSCs and osteoblasts significantly, and accelerated cell ageing, but did not affect the function of osteoclasts, resulting in loss of bone mass and osteoporosis. Saeed et al^122^ found that the total bone mineral content and BMD decreased by 13% and 23%, respectively, after the *Tert* gene was knocked out in mice for 32 weeks. Li et al^101^ proved that *Tert*‐transfected MSCs could help enhance proliferation and osteogenic differentiation ability in osteoporosis patients, so as to improve both bone mass and BMD, representing an effective material for further treatment of osteoporosis with autologous transplantation of MSCs.

### 4.3 Targeted modification of MSCs

To improve the bone‐targeted efficacy of transplanted MSCs, targeted peptides that could transfer the MSCs to the bone surface, have been applied to modify MSCs. This new method of increasing the homing and retention of the MSCs to bone has been assessed in preclinical studies. Guan et al^123^ have exploited an approach to guide MSCs to the bone surface by combining a synthetic high‐affinity and specific peptidomimetic ligand (LLP2A) against integrin α4β1 on the MSC surface to alendronate (Ale), which has a high appetence for bone. They have shown that LLP2A‐Ale directs the transplanted MSCs to the bone to augment endogenous bone formation and bone mass. LLP2A‐Ale also prevented trabecular bone loss after peak bone acquisition was achieved or as a result of oestrogen deficiency.

Targeted peptide and gene modification can also be used in combination. Chen et al^124^ put forward a strategy that utilized the high appetency of a unique DSS6 peptide (six repetitive sequences of aspartate, serine and serine) for bone surfaces and employed this peptide as a novel targeting vehicle to deliver and retain *PDGFB* at the site of bone loss. The result revealed that a large augment in bone formation could be achieved by engrafting an extremely low level of MSCs in the OVX mice. Compared with other groups, ALP levels increased by approximately 75% and trabecular bone density increased massively in the *PDGFB‐*DSS6 group.

### 4.4 Co‐culture and co‐transplantation

Some studies have reported that abnormal activation changes in OVX‐MSCs might decrease their effects in the treatment of osteoporosis. Thus, it would be beneficial to develop a special in vitro co‐culture system to enhance the proliferation, homing and osteogenic differentiation ability of MSCs to improve their performance. Saito et al^125^ developed a new activator for BMMSCs called Wharton's jelly extract supernatant (WJS), using human umbilical cord extracts, which contained various physiologically active substances. The results showed the proliferation of OVX‐MSCs was increased by co‐culturing with WJS in vitro, which was identified to be profitable for cell therapy to secure a sufficient number of cells from bone marrow for transplantation in a relatively brief period. The BMMSCs homing ability to damaged tissues was also improved. This would greatly benefit therapeutic efficiency by promoting cell distribution. The fusion of osteoclasts and bone resorption was inhibited. Trabecular bone volume and thickness were improved significantly in vivo by transplanting WJS‐activated OVX‐MSCs, as observed using micro‐CT.

During bone formation, angiogenesis and osteogenesis are mutually interdependent.^126^ The application of endothelial progenitor cells (EPCs) has been shown to initiate and facilitate neovascularization.^127^ He et al^128^ demonstrated that co‐culture of BMP‐2‐modified MSCs and EPCs significantly increased the osteoblastic differentiation of MSCs and endothelial differentiation of EPCs in vitro, and co‐transplantation of both cells promoted the growth of new blood vessels and osteogenesis in vivo.

Increased research attention has concentrated on co‐localization of the MSCs and HSCs niches and their functional interdependence within the bone marrow. MSCs are key elements in the bone marrow niche, where HSCs regulate MSC fate through BMPs, and MSCs influence the mobilization of HSCs by secreting soluble factors.^129^ The efficacy of co‐transplantation of MSCs and HSCs was mainly reflected in the following three aspects: (a) Co‐transplantation of human MSCs and HSCs into an in utero model of human‐sheep intensified the long‐term engraftment of human cells in the bone marrow and peripheral blood of the animals.^130^ Noort et al^131^ reported that the engrafted cells could be enhanced 3‐4 times when low numbers of HSCs were co‐infused with MSCs. (b) Hematopoiesis reconstitution was promoted significantly.^132^ Jung et al^129^ indicated that when HSCs and MSCs were co‐transplanted in micropores of 3D calcium phosphate scaffolds, the HSC‐MSC co‐seeded graft yielded markedly increased vascular number and diameter 4 weeks after ectopic implantation in immunodeficient mice. A significant elevation in the expression of human OCN was also confirmed in the HSC‐MSC group compared with MSCs seeded without HSCs. (c) Human HSCs were promoted to differentiate into B‐lymphocytes, granulocytes and megakaryocyte in vivo,^132^ illustrating that co‐transplantation could reconstruct B‐cell immunity, which might be related to the recovery of immunoregulation.^132^

## 5 CLINICAL TRIALS

To date, clinical trials of MSC transplantation for osteoporosis have mainly focused on the application of autologous cells; however, no results have been reported (Table 3).

**TABLE 3:** Clinical trials of MSC transplantation for bone regeneration

Cell type	Phase, patients	Disease treated	Treatment method	Dose	Therapeutic outcomes	Clinical trail
BMMSCs	Phase I, n = 10	Osteoporosis, Spinal fractures	Intravenous injection of autologous BMMSCs that were fucosylated	Four patients enrolled will received a single dose of 2 × 10^6^ cells/kg and six patients enrolled received a single dose of 5 × 10^6^ cells/kg	Study is estimated to be completed in May 2020	NCT02566655
ASCs	Phase II, n = 8	Osteoporotic fractures	ASCs were seeded within a composite graft and transplanted back into the fracture site	Not reported	Terminated, no results	NCT01532076

Abbreviations: ASCs, adipose‐derived mesenchymal stem cells; BMMSCs, Bone marrow mesenchymal stem cells.

### 5.1 Autologous BMMSCs

Although both autologous and allogeneic BMMSC transplantation are theoretically feasible, based on preclinical animal experiments, immune rejection is an inevitable risk for allogeneic transplantation and should be taken consideration in clinical trials.^133^ Therefore, all of the ongoing clinical trials have chosen autologous transplantation. The Hospital Clinico Virgen de la Arrixaca in Spain conducted an uncontrolled, open‐label clinical trial in Phase I sponsored by Red de Terapia Celular. In this clinical trial, operator collected autologous BMMSCs from patients approximately 30 days before transplantation and cultured them under controllable manufacturing conditions to expand to the dose range. The BMMSCs were subjected to a course of fucosylation and then injected intravenously into patients with osteoporosis. Four patients enrolled received a single dose of 2 × 10^6^ cells/kg and six patients received a single dose of 5 × 10^6^ cells/kg. After 24 months, bone resorption, formation and metabolism were measured using biochemical indexes, BMD was measured using dual‐energy X‐ray absorptiometry, and bone structure was evaluated using tissue morphology (ClinicalTrials.gov Identifier: NCT02566655).

### 5.2 Autologous ASCs

In addition to bone marrow‐derived cells, ASCs have also been studied for clinical use because of the wide distribution and availability of adipose tissues. An interventional phase II clinical trial using human ASCs to treat individuals over 50 years old with proximal humeral fractures as a model for osteoporotic fracture has been conducted by the University Hospital in Basel, Switzerland. In this study, ASCs were wrapped around hydroxyapatite microgranules embedded in a fibrin gel to allow cellularized composite graft augmentation. Clinical and/or radiological follow‐up was performed at 6, 9 and 12 months after operation. Functional assessment was performed at 6 weeks, and 6 and 12 months after operation using the Constant score and the Quick DASH score (disabilities of arm, shoulder and hand). However, the trial was terminated and no results were reported (NCT01532076).

## 6 CHALLENGES IN CLINICAL TRANSFORMATION

Although preclinical experiments using MSCs to treat osteoporosis have been established for years with positive effects and recognized mechanisms, there are still many challenges and hurdles to be faced in the process of clinical transformation, including safety issues, transplant efficiency and standardization of proliferation and the manufacturing processes.

### 6.1 Safety issues

First, the self‐renewal ability of MSCs carries an inherent risk of tumour formation. MSCs are directly involved in tumour progression and metastasis by changing the biological characteristics of cancer cells, regulating immune status and promoting angiogenesis.^134^ The results of a meta‐analysis of 39 relevant animal experiments by Li et al^135^ showed that MSC transplantation increased the incidence and quantity of tumour metastasis in an experimental tumour model. To date, there have been at least four clinical trials using MSCs to treat cancer; however, no results have been reported (NCT02008539, NCT02530047, NCT02068794, NCT01983709). Therefore, the relationship between MSC transplantation and tumours remains to be further clarified. The second risk is the potential formation of thrombus after cell transplantation. In a clinical trial of intravenous administration of expanded allogeneic ASCs in refractory rheumatoid arthritis, 141 side effects were reported in 53 patients, including one fatal case of lacunar cerebral infarction.^136^ Although the team believed that ASCs injection might have induced this infarction, the mechanism of this case remains unclear and requires confirmation. Besides, MSC transplantation might disrupt bone mineral metabolism. The volume of MSCs continues to shrink and become fragmented, accompanied by the release of exosomes, apoptotic bodies and cell fragments, which contain a large number of cytokines and disturb the local microenvironment.^137^ Kang et al^138^ and Beak et al^139^ reported that cell transplantation would contribute to bone mineral metabolism disorder in the short term and then lead to bone formation disorder and increased bone resorption. Thirdly, passaged MSCs often inevitably undergo phenotypic, functional, and more importantly, genetic changes,^140^ leading to unpredictable safety issues compared with the primary cells. In addition, their osteogenic potential is usually impaired, while their adipogenic potential may be preserved in passaged MSCs, suggesting that only MSCs at early passages would be effective for osteogenic differentiation.^140^

### 6.2 Transplant efficiency

A majority of MSCs were trapped inside the lungs following intravenous infusion, which was termed the pulmonary first‐pass effect.^141^ After the MSCs were concentrated in the lungs after intravenous injection, 1‐2.7% of the MSCs migrated to each organ, of which less than 1/8 of the MSCs homed to the bone marrow.^142^ Huang et al^143^ used an in vivo imaging system (IVIS) to observe the number of MSCs homing at different time slots after transplantation. They found that after intravenous injection, MSCs were initially retained in lungs for around 8‐9 days and then gradually remigrated to the fracture site. It was also reported that intra‐bone marrow injected MSCs could rapidly home to damaged bone tissue; however, the apoptosis rate was high, and less than 3% remained at the fracture site after 5 weeks.^143^ Fischer et al^141^ confirmed that the ability of MSCs to pass through pulmonary microvessels was related to their adhesion and deformation ability by transplanting different sizes of MSCs intravenously. Their results proved that infusion via two boluses increased pulmonary MSC passage compared with single bolus administration. The results explained why a single infusion of MSCs benefited transiently, and subsequent infusions with the same donor‐MSCs helped to maintain the beneficial effects.^144^ Furthermore, following transplantation of primary MSCs that had been cultured for only 24 hours, the homing capacity was reduced to 10%, while after transplantation of 48 hour‐cultured primary MSCs, no cells were detected in the target organs.^145^

### 6.3 Standardization of the manufacturing process

First, the performance and immunogenicity of MSCs from different tissue sources are different, and each has its own advantages and disadvantages. There is still controversy surrounding which tissue stem cells should be extracted from. Another problem is the standardization of MSC growth and their functional amplification, which is a mandatory objective of cell therapies. However, no unified standardized process has been proposed so far.^146^ Next, there is no unified standard for the MSC injection volume at present and 1‐5 × 10^6^/kg is commonly used in animal experiments.^147^ The last issue is that different administrations might lead to different therapeutic effects. Agata et al^148^ contrasted the safety and efficacy of intra‐bone marrow and intravenous administration of MSCs to treat OVX‐induced osteoporosis. They noticed that none of the mice died after intra‐bone marrow administration, whereas 22% of the mice died after intravenous administration. With reference to efficacy, intra‐bone marrow administration improved BMD by increasing both the bone mineral content and bone thickness, whereas intravenous administration improved BMD by increasing bone mineral content without affecting bone thickness. The results indicated that intra‐bone marrow administration of pure MSCs might be a safer and more effective method to treat osteoporosis.

## 7 PERSPECTIVES

Recently, growing attention has been focused on extracellular vesicles (EVs), which are secreted by MSCs^149^ and play a critical role in cell‐cell communication.^150^ Unlike MSCs, implanted EVs interact with their targets via signal transduction by docking at the plasma membrane of the target cell and/or via releasing the bioactive cargo upon fusion or endocytosis followed by fusing with the delimiting membrane of the endosomal compartment in bone‐remodelling microenvironment.^149^, ^151^ In addition to inhibiting the inflammatory response^152^ and promoting vascularization,^153^ which are similar to the effects of MSC transplantation, EVs have been found to promote bone formation by repairing the function of impaired MSCs^150^ and improving the activity of osteoblasts,^154^ suggesting that EV is a prospective therapeutic target for osteoporosis.^155^ Li et al^156^ and Chen et al^157^ explored the effects of EVs on the osteogenic, proliferation, and migration capabilities of BMMSCs in vitro, and demonstrated that EVs derived from ASCs promoted bone regeneration. Shen et al^158^ found that EVs fabricated from BMMSCs contained growth factors secreted by MSCs, and co‐culture with EVs in vitro increased the viability of osteoblast cells. Further in vivo experiments confirmed that injection of EVs mitigated OVX‐induced osteoporosis by reducing cell apoptosis and systemic inflammation, but increasing osteoblast numbers. Qi et al^153^ revealed that EVs stimulated bone regeneration and angiogenesis in critical‐sized calvarial defects in OVX rats and the effect of EVs increased with increasing concentration. Liu et al,^159^ Zhao et al^160^ and Zuo et al^161^ also proved that BMMSC‐derived EVs alleviated osteoporosis progression and bone loss, and elevated mineralized nodule and bone formation. Their data strongly suggested that EVs had an application prospect in the treatment of osteoporosis. Furthermore, Wang et al^162^ proved that EVs from human exfoliated deciduous teeth could enhance osteogenic differentiation in periodontal ligament stem cells, furnishing new insights into the application of EVs in periodontitis‐induced bone defect therapy.

Moreover, EVs have the following advantages compared with cell transplantation: (a) High security: With no expression of MHC proteins, EVs do not cause immune rejection, cell malignancies, and other problems.^163^ (b) Convenience: EVs can be stored at −20°C for a long time, making then easy to store and transport.^164^ (c) Stability: The outer lipid covers proteins, nucleic acids, and other contents to prevent them from being decomposed by body fluids.^165^ (d) The manufacturing process of EVs is easier to standardize than that of stem cells.^166^, ^167^ Although EVs might circumvent many of the problems associated with MSC transplantation and represent a future trend for osteoporosis treatment, the research is still at an early stage. Mechanistic research and clinical trials of stem cell therapy based on MSCs represent important foundations for research into the mechanisms of EVs and their clinical translation.

## CONFLICT OF INTEREST

The authors declare that they have no competing interests.

## AUTHOR CONTRIBUTIONS

JY searched the literatures and wrote the paper. PZ and XZ searched the literatures and revised the manuscript. LL and ZY conceived the review, revised the manuscript, final approval of the manuscript and financial support. All authors read and approved the final version of the manuscript.

## CONSENT FOR PUBLICATION

All authors agree to submit the manuscript for consideration for publication in the journal.

## Data Availability

Data sharing is not applicable to this article as no new data were created or analysed in this study.
